# Intraspecific Diversity Regulates Fungal Productivity and Respiration

**DOI:** 10.1371/journal.pone.0012604

**Published:** 2010-09-07

**Authors:** Anna Wilkinson, Martin Solan, Andrew F. S. Taylor, Ian J. Alexander, David Johnson

**Affiliations:** 1 Institute of Biological and Environmental Sciences, University of Aberdeen, Aberdeen, United Kingdom; 2 Oceanlab, University of Aberdeen, Newburgh, United Kingdom; 3 Macaulay Land Use Research Institute, Aberdeen, United Kingdom; University of Oxford, United Kingdom

## Abstract

Individuals and not just species are key components of biodiversity, yet the relationship between intraspecific diversity and ecosystem functioning in microbial systems remains largely untested. This limits our ability to understand and predict the effects of altered genetic diversity in regulating key ecosystem processes and functions. Here, we use a model fungal system to test the hypothesis that intraspecific genotypic richness of *Paxillus obscurosporus* stimulates biomass and CO_2_ efflux, but that this is dependent on nitrogen supply. Using controlled experimental microcosms, we show that populations containing several genotypes (maximum 8) of the fungus had greater productivity and produced significantly more CO_2_ than those with fewer genotypes. Moreover, intraspecific diversity had a much stronger effect than a four-fold manipulation of the carbon:nitrogen ratio of the growth medium. The effects of intraspecific diversity were underpinned by strong roles of individuals, but overall intraspecific diversity increased the propensity of populations to over-yield, indicating that both complementarity and selection effects can operate within species. Our data demonstrate the importance of intraspecific diversity over a range of nitrogen concentrations, and the need to consider fine scale phylogenetic information of microbial communities in understanding their contribution to ecosystem processes.

## Introduction

Community composition is strongly influenced by the genetic diversity of its constituent individuals and so we must consider how variation in genotypes, and not just species, in a community may contribute to ecosystem functioning [1]. Phenotypic variation within species can be substantial [2], and when this variation is expressed through ecologically important traits, intraspecific diversity becomes an important component of biodiversity [1]. In the few studies undertaken in plant communities, increasing genotypic richness of populations led to parallel effects on productivity [3], [4]. Intraspecific diversity may also have indirect effects, by interacting with species diversity. This may occur, firstly, when the presence of a particular genotype has a “disproportionate” effect on some key process that affects species diversity, *e.g*. litter decomposition rates in populations of *Populus* [5]. Secondly, it may be seen through differential effects on competition with other species, *e.g. S. altissima* interacting with invasive herbaceous plants [6].

Biodiversity theory is largely underpinned by observations and manipulations of plant communities. Whether these patterns are seen in microbial systems is largely unknown; moreover, because the phylogenetic and physiological diversity, abundance, biomass, and distribution of microorganisms is considerably greater than in plants and animals, current ecological theory is likely to be of limited value to microorganisms [7]. A key challenge in ecology, therefore, is to determine if the effects of biodiversity seen in plant and animal communities are also seen in soil microbial systems [8]. This is important, not just from the perspective of developing ecological theory, but because soil microorganisms play crucial roles in regulating global carbon (C) and mineral nutrient cycles.

Experimental manipulation has revealed the importance of species diversity of a number of functionally important fungal groups [9]–[11]. One factor contributing to the range of responses seen is likely to be the wide variation in the functional attributes of individuals. Evidence from *ad hoc* observations of genotypes of ectomycorrhizal (ECM) fungi in pure culture experiments show that they can differ markedly in traits like organic nitrogen (N) utilisation [12], N use efficiency [13] and growth rates [14] that have a direct impact on rates of nutrient cycling. These findings indicate that the performance of particular genotypes of ECM will differ depending on the availability of N. The availability of N may therefore be one factor that influences the extent to which genotypic richness regulates the performance of populations. Recent work has also shown that genotypes of different *Paxillus* species vary considerably in their ability to form mycorrhizas with birch [15]. Pure culture experiments in which the C∶N ratio of the growth medium was manipulated by fixing the N content demonstrated that both ECM fungal species and genotypes exhibited marked differences (up to three times) in their growth and respiratory responses to C supply [14]. These results suggest that C∶N ratio is likely to interact with fungal genotypic diversity and affect the response of populations to intraspecific richness. However, what these *ad hoc* observations of genotypic diversity have failed to consider is the potential importance of intraspecific richness. Given the large range of functional attributes seen in individual isolates of fungi, we predict that manipulation of intraspecific diversity of ECM fungal populations will have quantifiable effects on the overall performance of the population.

Here, we test the hypothesis that intraspecific genotypic richness of fungi stimulates biomass and CO_2_ efflux, but that this is dependent on N supply. We established a gradient of intraspecific richness of 1, 2, 4 and 8 genotypes of an ECM fungus (*Paxillus obscurosporus*) growing in pure culture using an established experimental design [10], in which all of the single genotypes were represented, to give a total of 15 unique treatments. We chose *P. obscurosporus* because closely related species have been demonstrated to perform well at high N availability [16]; we therefore predict that intraspecific richness will be less important in regulating biomass and CO_2_ efflux at high N availability.

## Results

**Figure 1:**
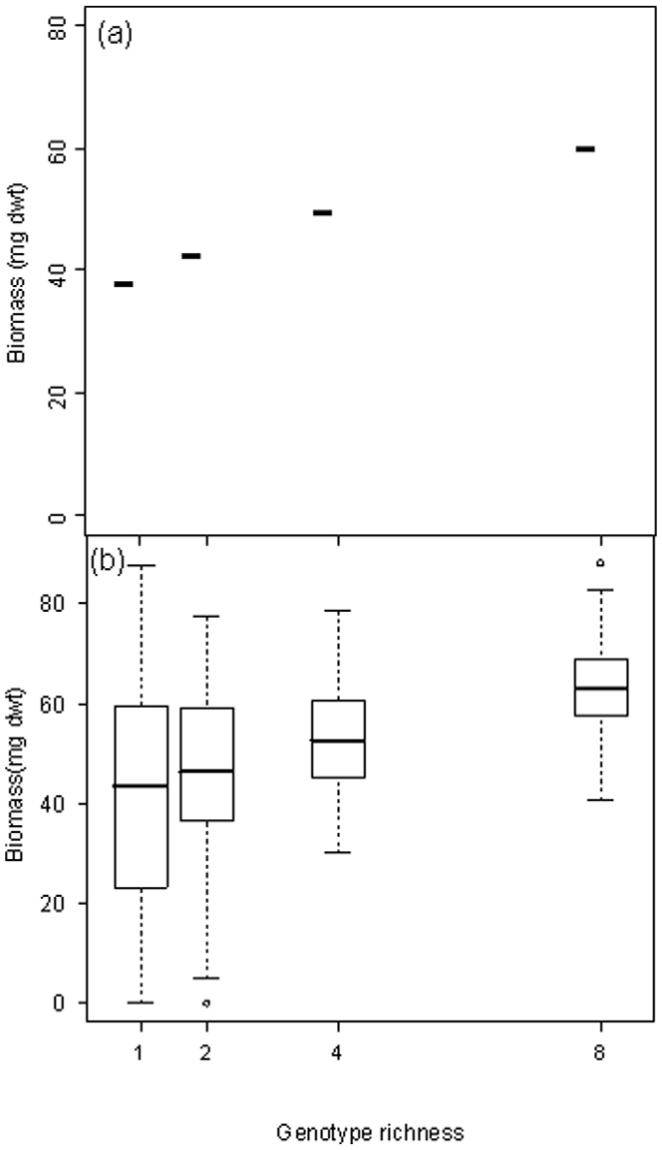
The effect of genotypic richness of the fungus *P. obscurosporus* on population biomass. In (a), horizontal bars represent predicted values from the optimal regression model. Genotypic richness was the most important factor influencing fungal biomass (L-ratio = 33.02, d.f. = 7, *P*<0.001), followed by a marginal effect of C∶N ratio (Figure S2). In (b), the horizontal bars represent predicted median values from the optimal regression model, vertical dashed lines represent the spread of the data, the upper and lower parts of the box indicate the 75% and 25% quartile, and circles are outlying values.

**Figure 2:**
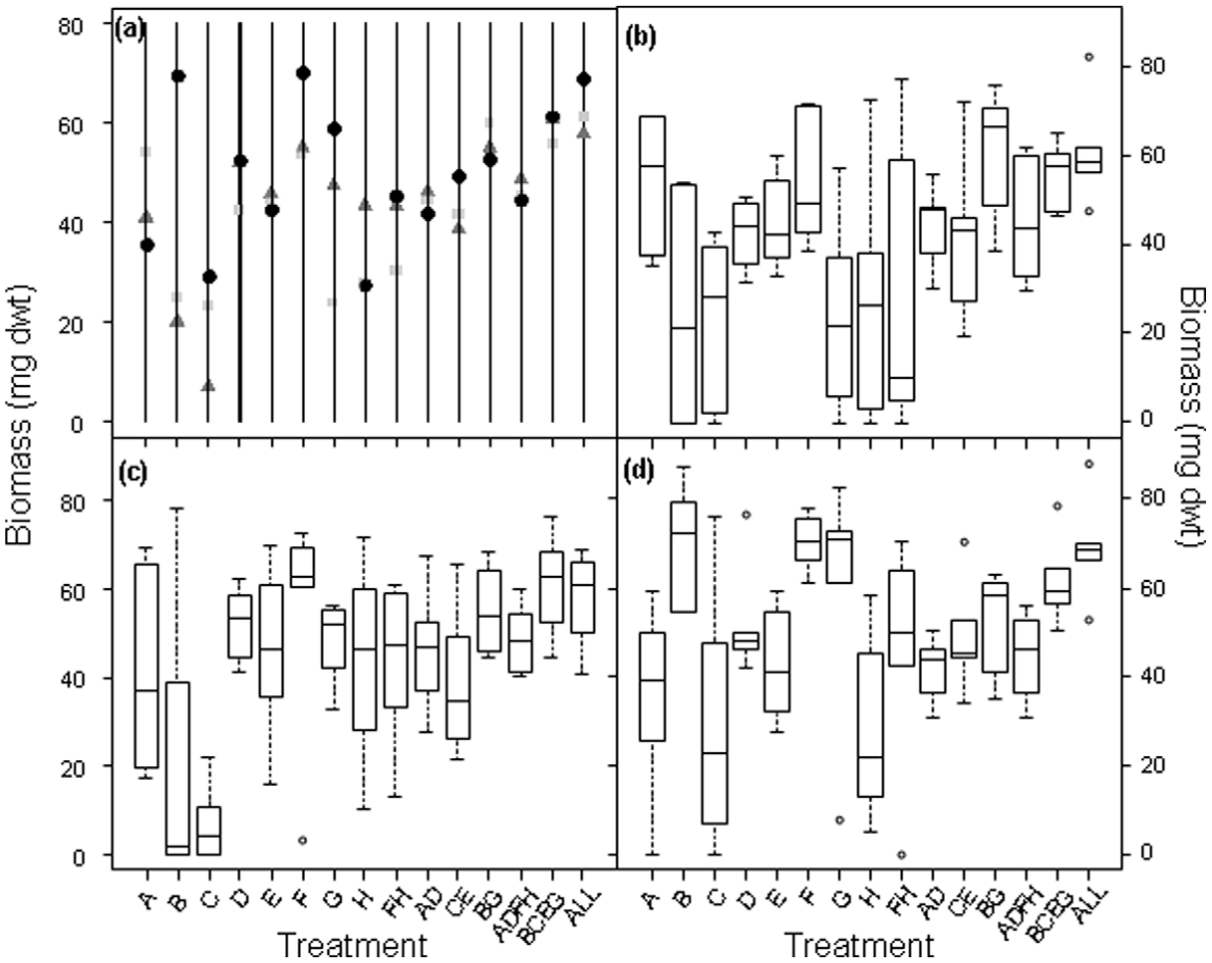
The effect of treatment identity and its interaction with substrate C∶N ratio on fungal biomass. In (a), the symbols are the predicted values from the optimal regression model for each of the 15 unique fungal populations for each C∶N ratio (10∶1 = light grey square; 20∶1 = dark grey triangle; 40∶1 = black circle). Each letter (A–H) represents an individual genotype and combinations describe the particular diversity treatments (Table S1). Treatment identity was the most important factor influencing fungal biomass (L-ratio = 133.3, d.f. = 48, *P*<0.001) followed by C∶N ratio (L-ratio = 49.9, d.f. = 60, *P* = 0.013). The spread of the raw data (lines interpreted as for Figure 1) are shown for C∶N ratios of (b) 10∶1, (c) 20∶1 and (d) 40∶1.

In all microcosms, the strains were observed to grow and survive for the duration of the experiment. We found that intraspecific richness had a significant positive effect (L-ratio = 33.0, *P*<0.001) on fungal biomass (Figure 1; model 1 in Table 1). The biomass of treatments with a genotype richness of 4 and 8 was significantly greater than those with just one genotype (t = 4.03, *P* = 0.0001 and t = 6.11, *P* = 0.0001 respectively; Table S2). The most striking effect was seen in the 8 genotype treatment where biomass was approximately 50% greater than the single genotype treatments. In contrast, C∶N ratio had a weaker, marginal effect (L-ratio = 4.87, *P* = 0.088; Table 1 and S3) on fungal biomass, and did not interact with genotypic richness. The effects of intraspecific richness were underpinned by strong effects of individuals (Figure 2; L-ratio = 133.3; *P*<0.001; Table 1) and an interaction with C∶N ratio (L-ratio = 49.9; *P* = 0.013). The biomass of microcosms comprising single genotypes (Figure 2, genotype treatments A–H) was variable and ranged from 7 to 70 mg dwt. Some individuals produced consistently less (e.g. genotype treatment C produced less than genotype treatments A, D, E and F; model 2 in Table 1) or more biomass than others (e.g. genotype treatment F produced more than in treatments B, C, G and H; model 2). Increasing intraspecific richness also appeared to reduce the variation in response to substrate C∶N ratio (Figure 2). For example, the biomass of genotype treatments B and G ranged from 20 to 70 mg dwt in response to the three C∶N ratios (combined coefficient of variation (CV)  = 71.2%), but in combination (treatment BG), their biomass ranged from only 52 to 60 mg dwt (CV = 22.2%). Fungal biomass followed an upward trend when the genotypes were grown in mixture, but in many cases the productivity of particular combinations could not be predicted from that of the constituents when in monoculture, indicating non-additive effects. For example, at a C∶N of 40∶1, the mean biomass of treatment CE was greater than both monocultures C and E.

**Table 1:** Summary of the statistical analyses for the six GLS models (models 1 to 6).

	Response	Factors				Variance covariates	df* P*-value
		GR	TID	C∶N ratio	2 way interaction		
**Model 1**	Biomass	33.07<0.001	-	4.880.088	-	GR	13<0.001
**Model 2**	Biomass	-	133.3 48<0.001	49.9600.013	48.1620.001	TID * C∶N ratio	46<0.001
**Model 3**	CO_2_ efflux	38.07<0.001	-	5.580.064	-	GR	130.0004
**Model 4**	CO_2_efflux	-	200.7 48<0.001	65.660 <0.001	58.762<0.001	TID * C∶N ratio	46<0.001
**Model 5**	DmaxBiomass	10.1120.007	-	5.2120.075	-	GR * C∶N ratio	10<0.001
**Model 6**	DmaxCO_2_ efflux	24.26 <0.001	-	12.960.045	14.0180.026	C∶N ratio	10<0.001

For each relevant factor, L-ratio, degrees of freedom (df) and *P*-value are presented. GR  =  genotypic richness; TID  =  treatment identity (i.e. the 15 unique populations within a given C∶N ratio). See methods and supporting information for details of models.

**Figure 3:**
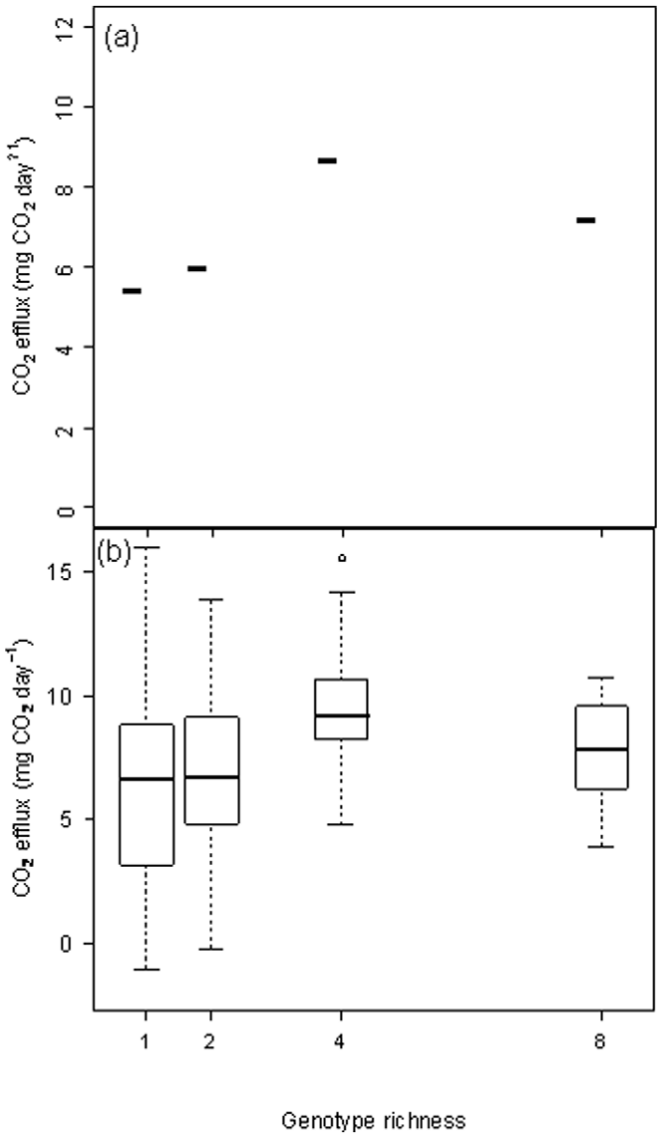
The effect of genotypic richness on CO_2_ efflux from microcosms containing populations of *P. obscurosporus*. Interpretation of bars and plots follows Figure 1. Genotypic richness was the most important factor influencing CO_2_ efflux (L-ratio = 38.0, d.f. = 7, *P*<0.001), followed by a marginal effect of C∶N ratio (Figure S3).

**Figure 4:**
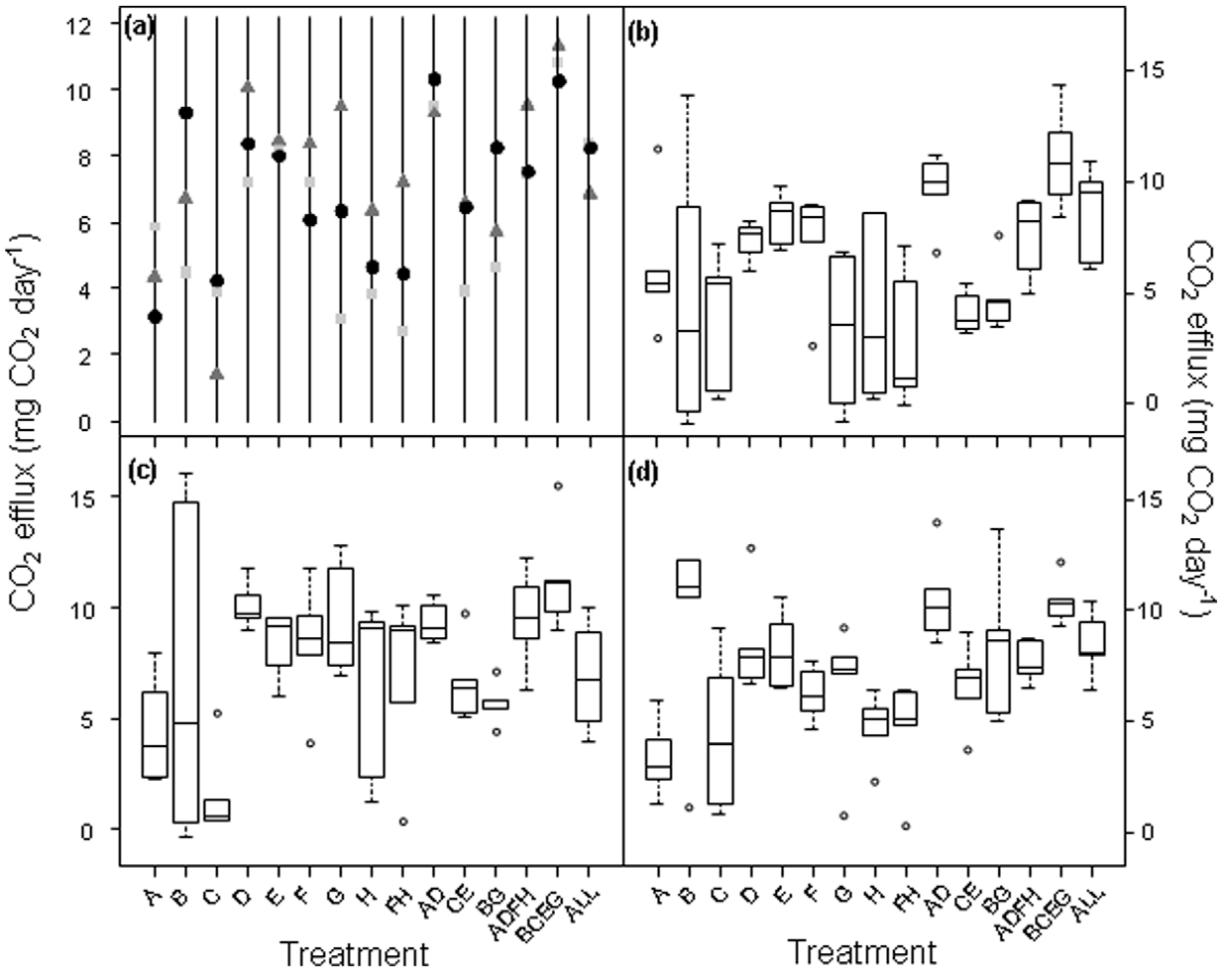
The effect of treatment identity and its interaction with substrate C∶N ratio on CO_2_ efflux. In (a), the symbols are the predicted values from the optimal regression model for each of the 15 unique fungal populations for each C∶N ratio (10∶1 = light grey square; 20∶1 = dark grey triangle; 40∶1 = black circle). Each letter (A–H) represents an individual genotype and combinations describe the particular diversity treatments (Table S1). Treatment identity was the most important factor influencing CO_2_ efflux (L-ratio = 200.7, d.f. = 48, *P*<0.001) followed by C∶N ratio (L-ratio = 65.6, d.f. = 60, *P*<0.001). The spread of the raw data (lines interpreted as for Figure 1) are shown for C∶N ratios of (b) 10∶1, (c) 20∶1 and (d) 40∶1.

Differences in CO_2_ efflux between treatments were similar at each sampling time, but maximum efflux at all C∶N ratios occurred between day 15 and 20 (Figure S1), so all statistical analyses used data from day 20. The pattern of CO_2_ efflux was similar to that seen for biomass, with respiration largely driven by intraspecific richness (Figure 3; L-ratio = 38.0, *P*<0.001; Tables 1 and S4) followed by a weaker marginal effect of C∶N ratio (L-ratio = 5.5, *P* = 0.064; Tables 1 and S5). The greatest CO_2_ efflux was in the 4-genotype treatment and was ∼60% greater than that of single genotypes (Figure 3; t = 6.61, *P*<0.001; Table S4). In the 8-genotype treatment, CO_2_ efflux was also significantly greater than in the single genotype treatments but the effect was slightly weaker (t = 2.76, *P* = 0.006; Table S4). Intraspecific richness effects on CO_2_ efflux were underpinned by strong identity effects (Figure 4; L-ratio = 200.7, *P*<0.001; Table 1) interacting with C∶N ratio (L-ratio = 65.6, *P*<0.001). In monoculture, genotype treatment C produced significantly less CO_2_ than genotype treatments D–F, while genotype treatments D and E produced significantly greater quantities than treatments C, G and H.

**Figure 5:**
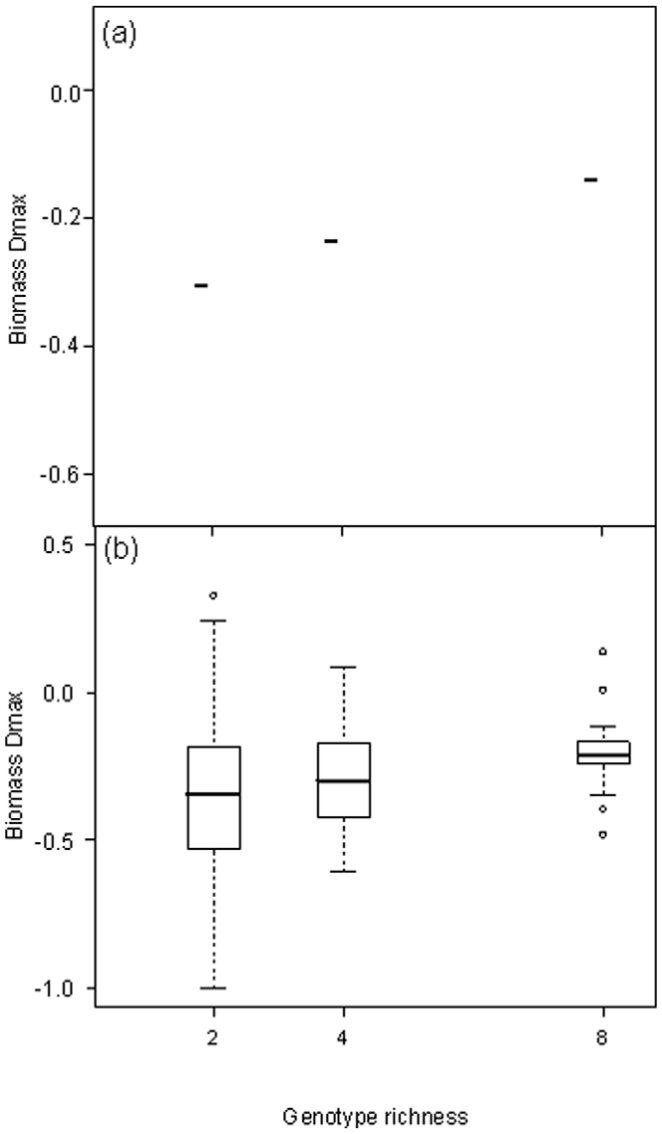
Transgressive overyielding (*D*_max_) of fungal biomass in response to genotype richness. Interpretation of bars follows Figure 1. Genotypic richness was the most important factor (L-ratio = 10.1, d.f. = 12, *P* = 0.007), followed by a marginal effect of C∶N ratio (L-ratio = 5.2, d.f. = 12, *P* = 0.074).

**Figure 6:**
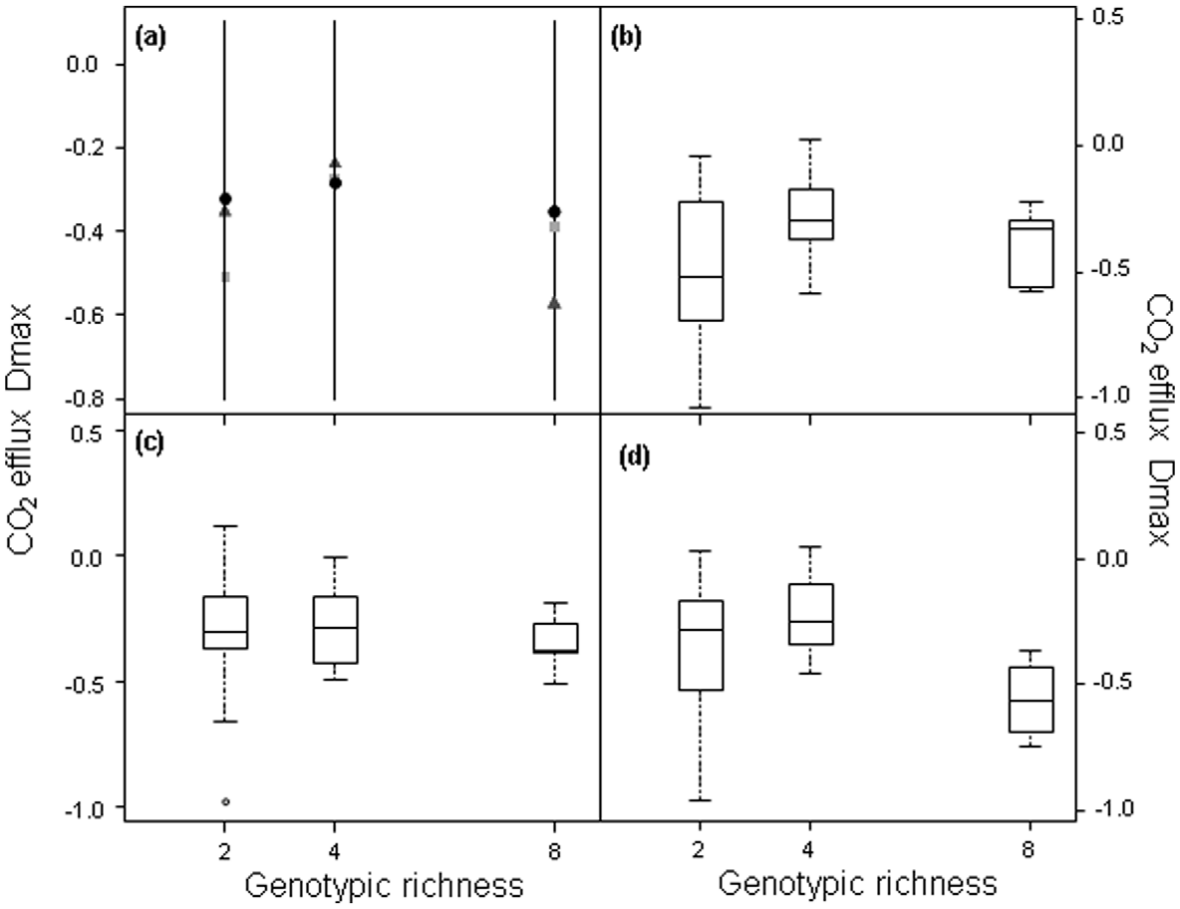
Transgressive overyielding of CO_2_ efflux in response to genotypic richness and substrate C∶N ratio. The symbols are the predicted values from the optimal regression model for genotype richness levels 2, 4 and 8 for each C∶N ratio (10∶1 = light grey square; 20∶1 = dark grey triangle; 40∶1 = black circle). Genotypic richness was the most important factor (L-ratio = 24.2, d.f. = 6, *P*<0.001), followed by C∶N ratio (L-ratio = 12.9, d.f. = 6, *P* = 0.044). The spread of the raw data for each level of genotypic richness are shown for C∶N ratios of (b) 10∶1, (c) 20∶1 and (d) 40∶1. Interpretation of bars and lines follows Figure 1.

We found that for biomass, transgressive overyielding (*D*_max_) was always <0, but there was a significant propensity for *D*_max_ to increase with intraspecific richness (Figure 5, L-ratio = 10.1, *P* = 0.007; Tables 1 and S6) and, marginally, to decrease with substrate C∶N ratio (L-ratio = 5.2, *P* = 0.075; Tables 1 and S7). For CO_2_ efflux, *D*_max_ was also <0, but it was dependent on intraspecific richness and an interaction with substrate C∶N (Figure 6, L-ratio = 24.2, *P*<0.0001; Tables 1 and S7), so that the maximum value of *D*_max_ did not necessarily occur in treatments with the greatest genotypic richness or lowest C∶N ratio. Although genotypic richness significantly increased the tendency to over-yield, in mixture treatments *D*_max_ was <0, indicating that most populations under-performed compared to outcomes predicted from monoculture responses.

## Discussion

We tested how increases in intraspecific diversity of fungi, here represented by the ECM fungus *P. obscurosporus*, and its interaction with N supply affected CO_2_ efflux and productivity, two key measures of ecosystem functioning. Our analyses consistently showed that intraspecific richness was the most important factor for both measured variables, but the effect was most apparent for biomass. These effects occurred despite there being little visual discernable difference in the morphology of the mycelia of the genotypes. It has been suggested that intraspecific variation in plants is likely to be most important when individuals exhibit phenological differences that are expressed through traits influencing ecosystem processes [1], [2]. This has led to several studies highlighting the potential for screening and classifying both species and individuals on the basis of trait variation [17], [18]. Whether such an approach can be taken with fungi remains to be tested, but there is clearly potential to undertake a broad screening exercise to identify the physiological and morphological traits that may contribute to regulation of key ecosystem processes. There is evidence that individuals of some fungal species can differ in their ability to produce mycelium [19], obtain N from organic sources [20] and colonise host plants [15], but the ubiquity of this variation and the importance of additive effects of different genotypes within natural communities is currently unknown. Our findings suggest that intraspecific richness is a key component of soil fungal biodiversity that may contribute to ecosystem functioning.

We would expect a four-fold difference in substrate C∶N ratio used in our study to have had significant impacts on the growth and respiratory activity of the fungal populations. Although we found that the C∶N ratio of the growth medium did lead to some changes in growth and CO_2_ efflux from the fungi, it is surprising that intraspecific richness had such consistent and strong effects. The data indicated that the biomass of genotypically rich populations tended to be more consistent across the three substrate C∶N ratios than genotypically poor populations (i.e. they often had smaller CV). This may in part be a result of the statistical averaging effect whereby the CV of biomass tends to decline with species richness [21]. It must also be remembered that *Paxillus* spp. are known to grow well where inorganic N is plentiful and so it is possible that other species may have a different response. One of the striking outcomes of plant species manipulation experiments highlighted in recent meta-analyses [22] is the large variation of biodiversity effects. Subsequent meta-analyses have provided evidence that intraspecific diversity can explain a large part of this variation [23]. In our study, the importance of intraspecific diversity over and above substrate C∶N ratio, which would normally be considered to be a central driver of ecosystem functioning, indicate that intraspecific diversity of fungi may contribute towards the wide range of variation seen in responses to species diversity. The study highlights the need to design experiments that enable partitioning of responses to both the effects of species and genotype.

When interpreted alongside the wide range of productivities in the single genotype treatments (Figures 2 and 4), the data from the mixtures suggest that selection effect (i.e. the increased likelihood of genotypically rich treatments containing a dominant individual) [24] is one factor in explaining the results. However, although *D*_max_ was always <0, there was a propensity for *D*_max_ to increase with intraspecific richness. This indicates that niche complementarity (both positive and negative effects) was also likely to be driving the responses, and similar responses have been seen in manipulations of bacterial species richness [25]. Both positive and negative effects of particular combinations are not surprising because recognition of ‘non-self’ by basidiomycete mycelia is known to trigger the release of volatiles, extracellular enzymes and secondary metabolites. In such circumstances, patterns of resource-use are changed leading to differences in colony morphology and growth rate [26]. In contrast to our expectation, biomass overyielding tended to decrease (albeit marginally) with decreasing N availability so that genotypically rich populations tend to perform worse than genotypically poor populations under low N supply. This under performance may have arisen from a combination of the increased competition and sub-optimal nutritional conditions for *P. obscurosporus*. Ultimately, a better understanding of the genetic differences underpinning findings like those we report here will come from sequencing entire genomes of individual isolates [27].

Whilst this study aimed to provide a test of fundamental ecological theory using soil and root dwelling fungi, the results should also be considered in the context of the natural environment. The range in C∶N ratio that we used is routinely seen in plant litter, which forms the main nutrient input to soils and the substrate most commonly colonised by ECM fungi. In addition, our use of a maximum of eight genotypes is broadly in line with current estimates of intraspecific diversity of ECM fungal species in forests, although such work is still in its infancy. For example, recent applications of molecular based methods have shown that individual pine seedlings can support several genotypes of *Hebeloma cylindrosporum*, and a typical m^2^ of forest floor contained ∼9 genotypes of this species [28], [29]. On the other hand, 9 genotypes of *Tricholoma matsutake* were found within a 100 m^2^ plot [30]. Although we recognise that our experimental system physically constrained the genotypes at scales that may not normally occur in nature, the duration of the experiment was such that populations were harvested before the surface of each microcosm was occupied. Nevertheless, the observation that some ECM fungal species can exhibit spatial structure [31] clearly suggests that space is one factor that could generate patterns and effects of biodiversity in natural systems.

Because many species of fungi, including *P. obscurosporus*, form mycorrhizal associations with trees, there is potential for intraspecific diversity to have important second-order effects on host plant productivity and growth. Indeed, because both the species [32] and genotype [33] composition of host plant communities can affect the species diversity of associated ECM fungi, there is potential for complex multi-trophic interactions between above and below ground diversity that warrant further investigation. It has also been shown that outcomes of interactions between different genotypes of bishop pine (*Pinus muricata*) and the ectomycorrhizal fungus *Rhizopogon occidentalis* were dependent on edaphic factors [34] indicating potential for the symbiosis to exhibit geographic selection mosaics across landscapes. Intraspecific diversity of soil fungi may therefore be a key component of “horizontal diversity” [35] that could contribute to the functioning of forest ecosystems. Further, our data demonstrate the application of ecological theory to fungi [8], despite the fact that phenotypic variation of individuals is much less obvious than in higher organisms like plants. The data also illustrate the potential importance of fungal intraspecific richness for key processes that have previously only been reported at the level of the species for mycorrhizal [10], [36] and saprotrophic fungi [37]. The data support recent calls to take into account fine scale phylogenetic information about microbial communities when assessing their contribution to ecosystem processes [38]. A crucial next step is to determine whether intraspecific richness interacts with species richness, and the relative contribution of each in regulating ecologically important processes.

## Materials and Methods

### Microcosms

A gradient of genotypic richness was created using 8 different strains (isolated from separate sporocarps and kindly donated by Dr. J. Hedh) of the ECM fungus *Paxillus obscurosporus* (see Table S1). The identity of the isolates was confirmed by sequence analysis of the internal transcribed spacer region of rDNA (Table S1). Isolates from this species have previously been determined to belong to a lineage I of the *Paxillus involutus* group [15]. Fifteen unique treatments were created of which 8 were single genotype monocultures (treatments A–H), 4 were mixtures of 2 genotypes (treatments FH-BG), 2 were mixtures of 4 genotypes (treatments ADFH and BCEG), and 1 comprised all genotypes (treatment ALL). The 2 and 4 genotype mixtures were drawn at random without replacement. The experiment used individual, gas-tight 500 ml glass Kilner jars containing 50 ml pH 5.5 sterile modified Melin Norkrans (MMN; [39]) solid growth media covered with sterile cellophane discs. Three levels of N availability were established in the media (C∶N ratios of 10∶1, 20∶1 and 40∶1) by holding C content constant and varying N content. The MMN media therefore contained 15 g l^−1^ agar, 5 g glucose l^−1^ as the C source and 0.900 g l^−1^, 0.450 g l^−1^ and 0.225 g l^−1^ (NH_4_)_2_HPO_4_ as the N source for the 10∶1, 20∶1 and 40∶1 C∶N ratio treatments, respectively). Inoculum plugs (3 mm diameter removed from the growing margins of colonies from identical MMN media) were transferred to the cellophane-covered agar in the Kilner jars. The cellophane prevents mycelium from penetrating into the media below, but also permits exchange of nutrients through it Eight fungal plugs placed at random in a uniform grid comprising two outer lines of three and an inner line of 2 were used in each treatment. Each microcosm jar had approximately equal amounts of inoculum at the start of the experiment, although it is possible that a small amount of variation in hyphal density could add to variation seen in the data. There were six replicates for each treatment (total number of microcosms = 15 diversity treatments ×3 N treatments ×6 replicates = 270). Each microcosm contained a vial of 10 ml 1 M NaOH to trap evolved CO_2_ (i.e. fungal respiration), and an additional series of uninoculated controls accounted for carbon accumulation through abiotic pathways. The microcosms were kept in the dark at 27°C. The NaOH samples were removed approximately every five days for 25 days and the total amount of CO_2_ produced during the experiments was determined by back-titration using a digital burette. After 25 days, the cellophane was removed from the Kilner jars and the total fungal tissue in each microcosm was scraped from it, dried, weighed and corrected for the weight of the initial inoculum.

### Statistical analysis

A generalized least squares (GLS) statistical mixed modelling approach was used [40], [41] to account for the unequal variance imposed by the experimental design using suitable variance-covariate functions. The fixed structure of the model was established by applying backward selection using the likelihood ratio (L-ratio) test obtained by Maximum Likelihood (ML). The numerical output of the minimal adequate model was obtained using REML estimation (Appendix S1) [42]. These analyses were all performed using the ‘nlme’ package (ver. 3.1) in the ‘R’ statistical and programming environment [43]. The statistical tests used cannot be applied directly to mean values with standard errors but instead relate to model predictions; these are therefore what we present in the main paper. However, the treatment means (±SEM) are also presented in Figures S4 and S5. To determine if genotypic combinations had positive effects on biomass and CO_2_ efflux, we compared biomass and respiration in the genotype combinations relative to the best performing monocultures (transgressive overyielding (*D*_max_); [44], [45]). *D*_max_>0 if a mixture of genotypes produces more biomass or more CO_2_ than its most productive (maximally yielding) constituent when in monoculture.

## Supporting Information

Appendix S1 Details of R code used for the six statistical models.
(0.03 MB DOC)

Figure S1 Mean CO_2_ efflux (mg CO_2_ day-1) from microcosms over a 25 day time period for each C∶N ratio treatment level (± SE). As it is important to ensure that any genotypic effects observed were not related to increasing or declining phases of population growth, all statistical analyses were performed on data from when the population exhibited peak respiration, i.e., day 20 throughout the C∶N range.
(0.04 MB DOC)

Figure S2 Box plots showing the marginal effect of C∶N ratio on fungal biomass (mg dwt) production (L-ratio = 4.88, d.f. = 8, P = 0.088). Horizontal bars represent the median, vertical dashed lines represent the spread of the data and the upper and lower parts of the box indicate the 75% and 25% quartile.
(0.03 MB DOC)

Figure S3 Box plots showing the marginal effect of C∶N ratio on fungal CO_2_ efflux (mg CO_2_ day-1) (L-ratio = 5.50, d.f. = 8, P = 0.064). Horizontal bars represent the median, vertical dashed lines represent the spread of the data and the upper and lower parts of the box indicate the 75% and 25% quartile.
(0.03 MB DOC)

Figure S4 Mean biomass (±SE) of genotype treatments (1–15, see Table S1) grown on media with C∶N ratios of 10∶1 (upper panel), 20∶1 (middle panel) and 40∶1 (lower panel).
(0.19 MB DOC)

Figure S5 Mean CO_2_ efflux (±SE) of genotype treatments (1–15, see Table S1) grown on media with three C∶N ratios of 10∶1 (upper panel), 20∶1 (middle panel) and 40∶1 (lower panel).
(0.22 MB DOC)

Table S1 GenBank accession numbers and combinations of *Paxillus obscurosporus* genotypes used in the experiment. Fungi were isolated from sporocarps collected from Skäne, Lund, Sweden.
(0.04 MB DOC)

Table S2 Coefficient table for model 1 (GR). Biomass coefficients (±SE), t and P values (in parentheses) among different levels of genotype richness (GR) are presented. Intercept ± SE (when baseline  =  GR1): 38.60±2.58, t = 14.98, p<0.001.
(0.03 MB DOC)

Table S3 Coefficient table for model 1 (C∶N ratio). Biomass coefficients (±SE), t and P values (in parentheses) among different levels of substrate C∶N ratio are presented. Intercept ± SE (when baseline  =  C∶N ratio of 10∶1): 38.60±2.58, t = 14.98, p<0.001.
(0.03 MB DOC)

Table S4 Coefficient table for model 3 (GR). CO_2_ efflux coefficients (±SE), t and P values (in parentheses) among different levels of genotype richness (GR) are presented. Intercept ± SE (when baseline  =  GR1): 5.55±0.42, t = 13.22, p<0.001.
(0.03 MB DOC)

Table S5 Coefficient table for model 3 (C∶N ratio). CO_2_ efflux coefficients (±SE), t and P values (in parentheses) among different levels of substrate C∶N ratio are presented. Intercept ± SE (when baseline  =  C∶N ratio of 10∶1): 5.55±0.42, t = 13.22, p<0.001.
(0.03 MB DOC)

Table S6 Coefficient table for model 5 (GR). Biomass overyielding (Dmax) coefficients (±SE), t and P values (in parentheses) among different levels of genotypic richness (GR) are presented. Intercept ± SE (when baseline  =  GR1): −0.30±0.05, t = 6.15, p<0.001.
(0.03 MB DOC)

Table S7 Coefficient table for model 5 (GR). Biomass Overyielding (*D*max) coefficients (±SE), t and P values (in parentheses) among different substrate C∶N ratios are presented. Intercept ± SE (when baseline  =  C∶N ratio of 10∶1): −0.30±0.05, t = 6.15, p<0.001.
(0.03 MB DOC)
