# Supplementary material for: Intraspecific Diversity Regulates Fungal Productivity and Respiration
Source: PLoS One. 2010 Sep 7;5(9):e12604. doi: 10.1371/journal.pone.0012604 (PMC2935373; doi:10.1371/journal.pone.0012604)
Supplement: Appendix S1 — Details of R code used for the six statistical models. (0.03 MB DOC) [file pone.0012604.s001.doc]

**Appendix S1**. Details of R code used for the six statistical models.

The form of the initial linear regression model with generalised least squares (GLS) estimation (incorporating variance covariates) for the effects of factors genotype richness (GR), treatment identity (TID) and C:N ratio of the substrate on different fungal responses:

RESPONSE ~ as.factor(GR/TID) + as.factor(C:N) + as.factor(GR/TID):as.factor(C:N),

weights = varIdent(form = ~ 1|as.factor(GR/TID)*as.factor(C:N)), method = "REML"

Minimal adequate models were as follows:

Model 1:

BIOMASS ~ as.factor(GR) + as.factor(C:N),

weights = varIdent(form = ~ 1|as.factor(GR)), method = "REML"

Model 2:

BIOMASS ~ as.factor(TID) + as.factor(C:N) +

as.factor(TID):as.factor(C:N),

weights = varIdent(form = ~ 1|as.factor(TID)*as.factor(C:N)), method = "REML"

Model 3:

RESPIRATION ~ as.factor(GR) + as.factor(C:N),

weights = varIdent(form = ~ 1|as.factor(GR)), method = "REML")

Model 4:

RESPIRATION ~ as.factor(TID) + as.factor(C:N) +

as.factor(TID):as.factor(C:N),

weights = varIdent(form = ~ 1|as.factor(TID)*as.factor(C:N)), method = "REML")

Model 5:

Dmax[BIOMASS] ~ as.factor(GR) + as.factor(C:N),

weights = varIdent(form = ~ 1|as.factor(C:N) * as.factor(GR)), method = "REML")

Model 6:

Dmax[RESPIRATION] ~ as.factor(GR) + as.factor(C:N) +

as.factor(GR):as.factor(C:N),

weights = varIdent(form = ~ 1|as.factor(GR)), method = "REML")
