# Supplementary material for: Intraspecific Diversity Regulates Fungal Productivity and Respiration
Source: PLoS One. 2010 Sep 7;5(9):e12604. doi: 10.1371/journal.pone.0012604 (PMC2935373; doi:10.1371/journal.pone.0012604)
Supplement: Figure S1 — Mean CO2 efflux (mg CO2 day-1) from microcosms over a 25 day time period for each C∶N ratio treatment level (± SE). As it is important to ensure that any genotypic effects observed were not related to increasing or declining phases of population growth, all statistical analyses were performed on data from when the population exhibited peak respiration, i.e., day 20 throughout the C∶N range. (0.04 MB DOC) [file pone.0012604.s002.doc]

**Figure S1.** Mean CO_2_ efflux (mg CO_2_ day^-1^) from microcosms over a 25 day time period for each C:N ratio treatment level (±SE). As it is important to ensure that any genotypic effects observed were not related to increasing or declining phases of population growth, all statistical analyses were performed on data from when the population exhibited peak respiration, i.e. day 20 throughout the C:N range.
