# Supplementary material for: Intraspecific Diversity Regulates Fungal Productivity and Respiration
Source: PLoS One. 2010 Sep 7;5(9):e12604. doi: 10.1371/journal.pone.0012604 (PMC2935373; doi:10.1371/journal.pone.0012604)
Supplement: Figure S3 — Box plots showing the marginal effect of C∶N ratio on fungal CO2 efflux (mg CO2 day-1) (L-ratio = 5.50, d.f. = 8, P = 0.064). Horizontal bars represent the median, vertical dashed lines represent the spread of the data and the upper and lower parts of the box indicate the 75% and 25% quartile. (0.03 MB DOC) [file pone.0012604.s004.doc]

**Figure S3.** Box plots showing the marginal effect of C:N ratio on fungal CO_2_ efflux (mg CO_2_ day^-1^) (L-ratio=5.50, d.f.=8, *P*=0.064). Horizontal bars represent the median, vertical dashed lines represent the spread of the data and the upper and lower parts of the box indicate the 75% and 25% quartile.
