# Supplementary material for: Intraspecific Diversity Regulates Fungal Productivity and Respiration
Source: PLoS One. 2010 Sep 7;5(9):e12604. doi: 10.1371/journal.pone.0012604 (PMC2935373; doi:10.1371/journal.pone.0012604)
Supplement: Figure S4 — Mean biomass (±SE) of genotype treatments (1–15, see Table S1) grown on media with C∶N ratios of 10∶1 (upper panel), 20∶1 (middle panel) and 40∶1 (lower panel). (0.19 MB DOC) [file pone.0012604.s005.doc]

**Figure S4.** Mean biomass (±SE) of genotype treatments (1-15, see table S1) grown on media with C:N ratios of 10:1 (upper panel), 20:1 (middle panel) and 40:1 (lower panel).
