# Supplementary material for: Intraspecific Diversity Regulates Fungal Productivity and Respiration
Source: PLoS One. 2010 Sep 7;5(9):e12604. doi: 10.1371/journal.pone.0012604 (PMC2935373; doi:10.1371/journal.pone.0012604)
Supplement: Table S1 — GenBank accession numbers and combinations of Paxillus obscurosporus genotypes used in the experiment. Fungi were isolated from sporocarps collected from Skäne, Lund, Sweden. (0.04 MB DOC) [file pone.0012604.s007.doc]

**Table S1.** GenBank accession numbers and combinations of *Paxillus obscurosporus* genotypes used in the experiment. Fungi were isolated from sporocarps collected from Skäne, Lund, Sweden.

| **Treatment identity** | **Genotype richness** | **Genotype combinations** | **GenBank accession** | **Culture collection reference** |
| --- | --- | --- | --- | --- |
| A | 1 | Genotype A | GU799607 | DJPax1 |
| B | 1 | Genotype B | GU799608 | DJPax2 |
| C | 1 | Genotype C | GU799609 | DJPax3 |
| D | 1 | Genotype D | GU799610 | DJPax5 |
| E | 1 | Genotype E | GU799611 | DJPax9 |
| F | 1 | Genotype F | GU799612 | DJPax10 |
| G | 1 | Genotype G | GU799613 | DJPax11 |
| H | 1 | Genotype H | GU799614 | DJPax12 |
| FH | 2 | Genotype F + H |  |  |
| AD | 2 | Genotype A + D |  |  |
| CE | 2 | Genotype C + E |  |  |
| BG | 2 | Genotype B + G |  |  |
| ADFH | 4 | Genotype A + D + F + H |  |  |
| BCEG | 4 | Genotype B + C + E + G |  |  |
| ALL | 8 | All genotypes |  |  |
