# Supplementary material for: Intraspecific Diversity Regulates Fungal Productivity and Respiration
Source: PLoS One. 2010 Sep 7;5(9):e12604. doi: 10.1371/journal.pone.0012604 (PMC2935373; doi:10.1371/journal.pone.0012604)
Supplement: Table S2 — Coefficient table for model 1 (GR). Biomass coefficients (±SE), t and P values (in parentheses) among different levels of genotype richness (GR) are presented. Intercept ± SE (when baseline = GR1): 38.60±2.58, t = 14.98, p<0.001. (0.03 MB DOC) [file pone.0012604.s008.doc]

**Table S2.** Coefficient table for model 1 (GR). Biomass coefficients (±SE), t and P values (in parentheses) among different levels of genotype richness (GR) are presented. Intercept ± SE (when baseline = GR_1_): 38.60 ± 2.58, t = 14.98, p < 0.001.

|  | **GR_1_** | **GR_2_** | **GR_4_** |
| --- | --- | --- | --- |
| **GR_2_** | 4.70 ± 3.01  1.56  (0.119) |  |  |
| **GR_4_** | 11.56 ± 2.87  4.03  (0.0001) | 6.87 ± 3.00  2.29  (0.0232) |  |
| **GR_8_** | 22.24 ± 3.64  6.11  (<0.0001) | 17.54 ± 3.75  4.68  (<0.0001) | 10.67 ± 3.64  2.94  (0.004) |
