# Supplementary material for: Intraspecific Diversity Regulates Fungal Productivity and Respiration
Source: PLoS One. 2010 Sep 7;5(9):e12604. doi: 10.1371/journal.pone.0012604 (PMC2935373; doi:10.1371/journal.pone.0012604)
Supplement: Table S3 — Coefficient table for model 1 (C∶N ratio). Biomass coefficients (±SE), t and P values (in parentheses) among different levels of substrate C∶N ratio are presented. Intercept ± SE (when baseline = C∶N ratio of 10∶1): 38.60±2.58, t = 14.98, p<0.001. (0.03 MB DOC) [file pone.0012604.s009.doc]

**Table S3.** Coefficient table for model 1 (C:N ratio). Biomass coefficients (±SE), t and P values (in parentheses) among different levels of substrate C:N ratio are presented. Intercept ± SE (when baseline = C:N ratio of 10:1): 38.60 ± 2.58, t = 14.98, p < 0.001.

| **C:N ratio** | **10:1** | **20:1** |
| --- | --- | --- |
| **20:1** | 1.50 ± 2.76  0.54  (0.589) |  |
| **40:1** | 5.78 ± 2.72  2.12  (0.035) | 4.29 ± 2.74  1.56  (0.120) |
