# Supplementary material for: Intraspecific Diversity Regulates Fungal Productivity and Respiration
Source: PLoS One. 2010 Sep 7;5(9):e12604. doi: 10.1371/journal.pone.0012604 (PMC2935373; doi:10.1371/journal.pone.0012604)
Supplement: Table S4 — Coefficient table for model 3 (GR). CO2 efflux coefficients (±SE), t and P values (in parentheses) among different levels of genotype richness (GR) are presented. Intercept ± SE (when baseline = GR1): 5.55±0.42, t = 13.22, p<0.001. (0.03 MB DOC) [file pone.0012604.s010.doc]

**Table S4**. Coefficient table for model 3 (GR). CO_2_ efflux coefficients (±SE), t and P values (in parentheses) among different levels of genotype richness (GR) are presented. Intercept ± SE (when baseline = GR_1_): 5.55 ± 0.42, t = 13.22, p < 0.001.

|  | **GR_1_** | **GR_2_** | **GR_4_** |
| --- | --- | --- | --- |
| **GR_2_** | 0.58 ± 0.49  1.20  (0.233) |  |  |
| **GR_4_** | 3.28 ± 0.50  6.61  (<0.0001) | 2.69 ± 0.53  5.08  (<0.0001) |  |
| **GR_8_** | 1.79 ± 0.65  2.76  (0.006) | 1.21 ± 0.68  1.79  (0.075) | -1.49 ± 0.68  -2.18  (0.030) |
