# Supplementary material for: Intraspecific Diversity Regulates Fungal Productivity and Respiration
Source: PLoS One. 2010 Sep 7;5(9):e12604. doi: 10.1371/journal.pone.0012604 (PMC2935373; doi:10.1371/journal.pone.0012604)
Supplement: Table S5 — Coefficient table for model 3 (C∶N ratio). CO2 efflux coefficients (±SE), t and P values (in parentheses) among different levels of substrate C∶N ratio are presented. Intercept ± SE (when baseline = C∶N ratio of 10∶1): 5.55±0.42, t = 13.22, p<0.001. (0.03 MB DOC) [file pone.0012604.s011.doc]

**Table S5.** Coefficient table for model 3 (C:N ratio). CO_2_ efflux coefficients (±SE), t and P values (in parentheses) among different levels of substrate C:N ratio are presented. Intercept ± SE (when baseline = C:N ratio of 10:1): 5.55 ± 0.42, t = 13.22, p < 0.001.

| **C:N ratio** | **10:1** | **20:1** |
| --- | --- | --- |
| **20:1** | 1.13 ± 0.47  2.39  (0.018 |  |
| **40:1** | 0.72 ± 0.47  1.55  (0.123) | -0.41 ± 0.47  -0.87  (0.386 |
