# Supplementary material for: Intraspecific Diversity Regulates Fungal Productivity and Respiration
Source: PLoS One. 2010 Sep 7;5(9):e12604. doi: 10.1371/journal.pone.0012604 (PMC2935373; doi:10.1371/journal.pone.0012604)
Supplement: Table S6 — Coefficient table for model 5 (GR). Biomass overyielding (Dmax) coefficients (±SE), t and P values (in parentheses) among different levels of genotypic richness (GR) are presented. Intercept ± SE (when baseline = GR1): −0.30±0.05, t = 6.15, p<0.001. (0.03 MB DOC) [file pone.0012604.s012.doc]

**Table S6**. Coefficient table for model 5 (GR). Biomass overyielding (*D*_max_) coefficients (±SE), t and P values (in parentheses) among different levels of genotypic richness (GR) are presented. Intercept ± SE (when baseline = GR_1_): -0.30 ± 0.05, t = 6.15, p < 0.001.

|  | **GR2** | **GR4** |
| --- | --- | --- |
| **GR4** | 0.068 ± 0.038  1.80  (0.075) |  |
| **GR8** | 0.164 ± 0.048  3.45  (0.001) | 0.096 ± 0.046  2.09  (0.039 |
