# Supplementary material for: Intraspecific Diversity Regulates Fungal Productivity and Respiration
Source: PLoS One. 2010 Sep 7;5(9):e12604. doi: 10.1371/journal.pone.0012604 (PMC2935373; doi:10.1371/journal.pone.0012604)
Supplement: Table S7 — Coefficient table for model 5 (GR). Biomass Overyielding (Dmax) coefficients (±SE), t and P values (in parentheses) among different substrate C∶N ratios are presented. Intercept ± SE (when baseline = C∶N ratio of 10∶1): −0.30±0.05, t = 6.15, p<0.001. (0.03 MB DOC) [file pone.0012604.s013.doc]

**Table S7.** Coefficient table for model 5 (GR). Biomass Overyielding (*D*_max_) coefficients (±SE), t and P values (in parentheses) among different substrate C:N ratios are presented. Intercept ± SE (when baseline = C:N ratio of 10:1): -0.30 ± 0.05, t = 6.15, p < 0.001.

| **C:N ratio** | **10:1** | **20:1** |
| --- | --- | --- |
| **20:1** | -0.066 ± 0.050  -1.303  (0.195) |  |
| **40:1** | -0.109 ± 0.048  -2.264  (0.026 | -0.044 ± 0.037  -1.17  (0.246) |
